# Automated Feedback After Internet-Based Depression Screening: Cost-Effectiveness Analysis of a Randomized Controlled Trial

**DOI:** 10.2196/68282

**Published:** 2025-12-23

**Authors:** Léon Gerardo Kreis, Hans-Helmut König, Franziska Sikorski, Bernd Löwe, Sebastian Kohlmann, Christian Brettschneider

**Affiliations:** 1 Department of Health Economics and Health Services Research, University Medical Center Hamburg-Eppendorf, Martinistr. 52, Hamburg, 20246, Germany, 49 40 7410 52445, 49 40 7410 40261; 2 Department of Psychosomatic Medicine and Psychotherapy, University Medical Center Hamburg-Eppendorf, Hamburg, Germany; 3 Department of General Internal Medicine and Psychosomatics, Heidelberg University Hospital, Heidelberg, Germany

**Keywords:** depressive disorder, screening, feedback intervention, online mental health intervention, cost-effectiveness, health economic evaluation

## Abstract

**Background:**

The clinical and cost-related consequences of internet-based depression screening, in combination with automated feedback, have been rarely investigated. We aimed to conduct a cost-effectiveness analysis of DISCOVER, a 3-armed, observer-masked, randomized controlled trial that focused on 2 versions of automated feedback interventions after internet-based depression screening.

**Objective:**

This study aimed to evaluate the cost-effectiveness of automated nontailored and tailored feedback interventions after internet-based depression screening from a societal perspective.

**Methods:**

Participants were recruited from the general population via traditional and social media. Participants who were undiagnosed but screened positive for depression on an online version of the Patient Health Questionnaire-9 (≥10 points) were randomized to automatically receive no feedback, nontailored feedback, or tailored feedback. The feedback interventions included the depression screening result, a recommendation to seek professional advice, and brief general information about depression. The tailored feedback was additionally framed according to the participants’ symptom profiles, treatment preferences, health insurance plans, and local residency. The time horizon was 6 months. The main outcome was the incremental cost-effectiveness ratio (ICER) from a societal perspective using quality-adjusted life years (QALY) based on the EuroQol-5D-5L. Cost-effectiveness acceptability curves were constructed. Furthermore, several sensitivity analyses and explorative subgroup analyses were conducted.

**Results:**

A total of 1012 participants (no feedback: n=343, 33.9%; nontailored feedback: n=338, 33.4%; and tailored feedback: n=331, 32.7%) were included. Differences in costs and effects were not statistically significant. However, ICER results indicated that both no feedback and tailored feedback exhibited dominance over nontailored feedback. The ICER of tailored feedback compared to no feedback was €109,730 per QALY (a currency exchange rate of €1=US $1.02 was applicable as of December 31, 2022), whereas both costs and QALYs were lower in tailored feedback. The cost-effectiveness probability of tailored feedback compared to no feedback ranged between 41% and 80%. Sensitivity analyses exhibited similar trends.

**Conclusions:**

Six months postintervention, feedback interventions had no statistically significant effect on costs from a societal perspective or on QALYs. Tailored feedback was associated with moderate cost-effectiveness probabilities compared to no feedback. Explorative subgroup analyses revealed subpopulations for which the interventions might be cost-effective.

## Introduction

Approximately 6% of adults experience depressive disorders worldwide [1]. As of 2019, depressive disorders were the 12th leading cause of disability-adjusted life years globally [2].

Depressive disorders are highly comorbid with other mental and somatic disorders, and studies suggest that preventing depression may causally prevent somatic diseases [3,4]. According to the World Health Organization, the COVID-19 pandemic has led to an increase of 27.6% in major depressive disorder cases in 2020, with women and young adults particularly affected [5]. Depressed individuals typically experience states of low mood, reduced motivation, loss of pleasure or interest, and feelings of guilt [6]. In addition, depression places a significant economic burden on society and is an important cause of sickness absence [7].

In primary care, depressive cases often remain undetected: only approximately 50% of cases are correctly diagnosed as such [8]. Depression screening can expose possible depressive cases and rule out nondepressive cases as a low-intensity intervention [9]. However, its effectiveness is much debated [9-11].

Internet-based depression screening is already broadly used; however, its economic consequences have not yet been investigated [12,13]. The web-based approach allows for automated tailoring of screening results to individual preferences, which in turn could motivate screened individuals to engage in guideline-based care. Online feedback might save costs compared to an in-person screening approach with a health care professional followed by a psychological or medical consultation. Moreover, web-based feedback is easily accessible 24/7 and can be linked to further digital offers.

The DISCOVER trial evaluated the efficacy of 2 targeted feedback interventions (tailored and nontailored feedback) after online depression screening [13]. The DISCOVER trial’s primary outcome was the change in depression severity. The interventions in the DISCOVER trial did not lead to a significant reduction in depressive symptoms. In addition, no statistically significant effects on secondary outcomes (eg, quality of life, help-seeking severity, anxiety, and somatic symptoms) were observed [13].

Although the interventions were not effective, conducting a health economic evaluation of such interventions can still provide relevant information beyond clinical effectiveness for several reasons. First, resources for health care are scarce. Hence, it is also important to evaluate the potential economic consequences of feedback after internet-based depression screening, as it might be a cost-saving strategy. Second, the effect measure of health economic evaluations goes beyond clinical effectiveness and uses a preference-based instrument to assess health-related quality of life, which takes multiple dimensions of health and the perspective of the general population into account. Third, health economic evaluations always consider two dimensions: costs and effects. Therefore, an ineffective intervention, which at the same time does not harm participants, could still be cost-effective when it leads to substantial cost reductions. The interventions in DISCOVER were designed to motivate individuals to engage in evidence-based depression care, which in turn might lead to increased costs. Therefore, it is important to analyze if costs in fact increased, suggesting the interventions led to a higher uptake of scarce health care resources without significant clinical effects, and thereby providing relevant information for decision makers and researchers. Finally, the cost-effectiveness of interventions that show no significant improvements in the primary outcome has been shown in previous literature [14,15].

Owing to the aforementioned reasons and as the DISCOVER trial collected data on both costs and effects during the 6-month follow-up period, a cost-effectiveness analysis (CEA) of targeted feedback interventions after internet-based depression screening is feasible and reasonable. To our knowledge, this is the first economic evaluation of feedback interventions following online depression screening.

## Methods

### Sample

The DISCOVER trial was an observer-masked, 3-armed randomized controlled trial (RCT) focusing on the effects of automated feedback interventions after internet-based depression screening on depression severity 6 months postintervention [16]. Participants were recruited from the general German population using social and traditional media campaigns, an online access panel, a newsletter, and internet search supported by a marketing company to ensure high representativeness of the German population. Further details on the recruitment and sample selection process are reported in the study protocol and the clinical effectiveness study [13,16]. Individuals were included based on a self-reported online assessment. Adults indicating at least *moderate* depressive symptoms (Patient Health Questionnaire-9 [PHQ-9] score ≥10) at baseline who had not received a depression diagnosis or treatment in the past year were included. In total, 5457 participants started screening, of which 4878 (89.39%) completed screening. Therefore, 1178 (24.15%) participants screened positive and had not received a depression diagnosis or treatment within the last year and were hence randomized into 1 of the 3 study arms, stratified by baseline depression severity [13]. Participants were included based on the intention-to-treat principle. However, 166 (14.09%) participants were excluded due to loss to follow-up, unavailability, or withdrawal of consent (Multimedia Appendix 1). This resulted in a final sample size of 1012 participants in the CEA.

### Interventions

Individuals who screened positive were randomized immediately after completing the internet-based depression screening into 1 of the 3 groups using a 1:1:1 allocation ratio. After screening, all participants were shown a *thank you* note on their screen. Participants allocated to the no feedback group did not receive any feedback on their screening result. Participants in the nontailored feedback group were presented with their depression screening result alongside the advice to seek professional help, brief general information on depression, and information on guideline-based treatment options. In the tailored feedback intervention, the information provided to the participants was adapted according to their responses in the baseline assessment; for example, the screening result and general depression information were presented according to the participants’ symptom profile, the advice to seek help was adapted according to individual specialist preferences, and treatment options were tailored according to participants’ health insurance provider. Further details on the interventions’ design can be found in the study protocol and the effectiveness study [13,16].

### Data Collection and Measures

#### Data Collection

The DISCOVER study protocol described the data collection process in detail [16]. Briefly, data were gathered online at 4 time points (T0-T3) and in 2 clinical telephone interviews. T0 and T1 represented the baseline assessment: T0 was the prerandomization assessment completed online between December 2020 and January 2022, and T1 occurred approximately 2 days after T0 and was the baseline postrandomization survey. Health-related quality of life (HRQL) and productivity losses were assessed at T0, and health care use was assessed at T1. T2 represented the 1-month follow-up, and T3 represented the 6-month follow-up.

Depression severity was measured using the PHQ-9 [17], HRQL was measured by completing the EuroQol-5D-5L (EQ-5D-5L), including the EuroQol visual analog scale (EQ VAS) [18,19]. The Somatic Symptom Scale-8 was used to estimate somatic symptom severity throughout the trial. Health care use, including medication intake, and productivity losses were assessed by means of a modified version of the Client Sociodemographic and Service Receipt Inventory [20]. In the DISCOVER trial, gender was assessed via participant self-report based on the question “What is your gender” with the options woman, man, or nonbinary. No data on sex assigned at birth were collected.

#### EuroQol-5D-5L

The EQ-5D-5L is a questionnaire to estimate HRQL in five dimensions: mobility, self-care, usual activities, pain or discomfort, and anxiety or depression [18,19]. Respondents answer for each dimension in 5 levels, ranging from 1 (no problems) to 5 (extreme problems), resulting in 1 of 3125 different health states (5⁵ combinations of 5 dimensions). The EQ-5D-5L features a visual analog scale (EQ VAS), ranging from 0 (worst imaginable health state) to 100 (best imaginable health state) as a measure of overall HRQL.

The EQ-5D-5L is a broadly applied HRQL measure, and its favorable psychometric properties have been demonstrated [21]. To assign preference-based values to responses on the EQ-5D-5L descriptive system, a value set representative of Germany was used [22]. EQ-5D-5L value scores range from −0.661 (worst imaginable health state) to 1 (best imaginable health state) [22].

#### Patient Health Questionnaire-9

The PHQ-9 is a measure of depression severity and recommended for screening, for example, by the US Preventive Services Task Force [10,17]. It consists of 9 questions regarding the frequency of depressive symptoms within the past 2 weeks, using a 4-point scale (0‐3) depending on the frequency of symptoms. The overall PHQ-9 score is the sum of points per item (range 0‐27), where depression severity increases with test score. Depression severity is indicated in five levels: (1) *minimal* (PHQ-9 score 0‐4), (2) *mild* (PHQ-9 score 5-9), (3) *moderate* (PHQ-9 score 10-14), (4) *moderately severe* (PHQ-9 score 15-19), and (5) *severe* (PHQ-9 score 20-27) depression [17]. The PHQ-9 is considered to have both high specificity and sensitivity, as well as superior psychometric characteristics [23]. The PHQ-9’s computerized version yields high validity and reliability compared to the paper-and-pen version [24].

#### Health Care Service Use

Use of health care services was estimated based on participants’ statements on an adapted version of the Client Sociodemographic and Service Receipt Inventory throughout the trial [20]. Data on use of inpatient, outpatient physician, and nonphysician services (eg, physiotherapy and massage), the use of pharmaceuticals, and formal and informal nursing care were collected.

#### Unit Costs

Most costs for health care services were calculated based on refined German standard unit costs [25]. For medication, we matched participants’ responses to pharmaceuticals, including their dosage, pack size, and pharmacy retail price. This was performed using the German pharmaceutical directory [26]. An overview of incorporated unit costs and their estimation methods can be found in Multimedia Appendix 2. We adjusted unit costs to prices of the reference year 2022 using the consumer price index [27]. A currency exchange rate of €1=US $1.02 was applicable as of December 31, 2022.

Productivity losses were estimated for both pre- and post-intervention periods based on the human capital approach. Indirect costs were assessed at T0, whereas direct costs from health care use were assessed at T1. As the interventions happened immediately after randomization at T0, we assigned the proportion of direct costs accruing between T0 and T1 to the posttreatment period rather than the pretreatment period. Total costs were the sum of direct and indirect costs. Intervention costs were not considered, as they are mostly fixed and hence decrease to a marginal level with higher use.

#### QALY Calculation

Quality-adjusted life years (QALY) were estimated based on the EQ-5D-5L index scores, assessed at T0 and T3 [22]. We assumed the change in HRQL between T0 and T3 was subject to a linear trend. A QALY is a 2D unit that takes into account HRQL and time. The term *1 QALY* represents 1 year at *perfect* HRQL. Therefore, QALYs during the follow-up period were calculated as described in Equation (1).


(1) $$QALY=\left[Index_{T0}+\;\left[\frac{Index_{T3}-Index_{T0}}{2}\right]*\left[\frac{180}{\Delta days\left(T0;T3\right)}\right]\right]*\left(\frac{1}{2}\right)$$

The adaptation of QALY to 180 days was applicable, as no included participants died during the trial. Costs and QALY were not discounted due to the short follow-up period of 6 months.

### Statistical Analysis

#### Missing Data

Missing data were a present phenomenon in the DISCOVER trial data. In total, 4.3% of all data points were missing. For variables with missing values, on average, 9% of the data were missing. Missing data were imputed using the multivariate imputation by chained equations [28]. Multiple imputation was performed to create 10 complete datasets with 927 participants each [28]. All statistical analyses were conducted using the programming language *R* (R Foundation for Statistical Computing) in the *RStudio 4.2.0* environment.

#### Base Case Analysis

As a base case, a societal perspective was assumed, including all available direct and indirect costs. The primary outcome of the CEA was QALY 6 months after the interventions. To test for successful randomization at baseline, pairwise comparisons between study arms regarding baseline sociodemographic characteristics, costs, HRQL, and depression severity were conducted.

In the first step, we used incremental cost-effectiveness ratios (ICERs) to estimate cost-effectiveness. The ICER describes the additional costs per extra unit of (health) effects gained due to an intervention. Pairwise ICERs were calculated as follows:


(2) $$ICER=\frac{\overset{-}{C_{IG}}-\overset{-}{C_{CG}}}{\overset{-}{E_{IG}}-\;\overset{-}{E_{CG}}}=\frac{\Delta \overset{-}{C}}{\Delta \overset{-}{E}}$$

where C represents costs, E represents effects, IG represents the intervention groups, and CG represents the control group.

We constructed cost-effectiveness acceptability curves (CEACs) to analyze the effect of willingness-to-pay (WTP) on cost-effectiveness probability, testing the robustness of ICER results. For this purpose, we performed monetary net benefit regressions (NBRs) considering different WTP margins [29]. We used a broad range of WTP margins between €0 and €160,000 per QALY with increments of €10,000. The theory behind the calculation of cost-effectiveness probabilities is described elsewhere [29].

NBRs were conducted as linear OLS regression models, where the net benefit was considered the dependent variable and the treatment dummy variable was considered the independent variable.

#### Sensitivity Analyses

To test the robustness of results, we conducted 5 deterministic sensitivity analyses.

In the first scenario (S1), we applied the EQ VAS score (divided by 100) to estimate QALYs.

In scenario 2 (S2), we analyzed cost-effectiveness for a dataset containing 4 formerly excluded participants (N_ext_=1016). Unreliable data points by these respondents were considered missing and imputed using the multivariate imputation by chained equations algorithm.

For scenario 3 (S3), we took the perspective of a health care payer, thus including all direct costs except informal care.

In scenario 4 (S4), the analyses were conducted on the sample used in the clinical effectiveness study (N_primary_=965 [13]). The sample used in S4 did not differ significantly from the base case sample used in this CEA (Multimedia Appendix 3).

In a fifth scenario (S5), we made use of PHQ-9 scores at T0, T2, and T3 to calculate depression-free days (DFDs) as an indicator of treatment effectiveness. In this scenario, we estimated the ICER in the unit (€ per DFD). The rationale behind this reliable and well-validated approach can be found elsewhere [30]. To calculate DFD in the pre- and post-intervention period, we applied linear interpolation to participants’ PHQ-9 scores. Changes in PHQ-9 scores between T0, T2, and T3 were assumed to be subject to a linear trend. According to an analysis from 2003, patients were willing to pay approximately 9% of their household income for freedom from depression symptoms [31]. We applied this value to the average monthly gross income of the reference year 2022 in Germany, yielding a WTP of €15 per DFD [32]. To gain a broader image of cost-effectiveness probabilities, we considered a range between €0 per DFD and €200 per DFD as possible WTP margins.

#### Explorative Subgroup Analyses

Additionally, we conducted several explorative subgroup analyses to gain further insights into the cost-effectiveness of the feedback interventions for different subpopulations of the sample. We conducted these analyses considering the categories gender, age (in terciles), depression severity at baseline (applying the PHQ-9), number of inhabitants in participants’ residency, if participants were diagnosed using the Structured Clinical Interview for DSM-5 Disorders at the postintervention baseline telephone interview, and if participants themselves believed at baseline that they had a depressive disorder.

### Ethical Considerations

The DISCOVER trial was reviewed and approved by the Ethics Committee of the Medical Chamber Hamburg in July 2019 (reference number: PV7039) and conducted according to the Declaration of Helsinki. All participants provided online informed consent before the online baseline assessment. Informed consent forms included information on study aims, procedures, privacy, and voluntary participation. Data were pseudonymized and securely stored on encrypted servers, ensuring confidentiality. For this CEA, data were used in accordance with the original consent provided. Participants were compensated with a 5€ voucher for the completion of each follow-up survey (T1, T2, and T3).

## Results

### Characteristics of the Study Population

A summary of baseline characteristics can be found in Table 1. The mean age of the sample was 37.50 (SD 14.09; range 18‐79) years. A total of 716 (71%) individuals were women, 287 (28%) were men, and 9 (1%) identified as nonbinary. The average depression severity was between *moderate* and *moderately severe* (mean PHQ-9 score 14.77, SD 4.00). At baseline, 56% (568/1012) of participants showed *moderate* depression severity, 30% (308/1012) indicated a *moderately severe*, and 13% (136/1012) showed a *severe* degree of depression. Baseline characteristics in the sample of this economic evaluation did not differ significantly compared to the sample of the effectiveness study (Multimedia Appendix 3) [13].

**Table 1.** Characteristics of the study population at baseline.

Characteristics	Overall (N=1012)	Nontailored feedback (n=338)	No feedback (n=343)	Tailored feedback (n=331)
Age (y), mean (SD)	37.50 (14.09)	38.25 (14.04)	36.98 (13.74)	37.27 (14.49)
Gender, n (%)				
Women	716 (70.8)	237 (70.1)	248 (72.3)	231 (69.8)
Men	287 (28.4)	98 (29.0)	92 (26.8)	97 (29.3)
Nonbinary	9 (0.9)	3 (0.9)	3 (0.9)	3 (0.9)
Living situation, n (%)				
With someone	677 (66.9)	232 (68.6)	228 (66.5)	217 (65.6)
Alone	335 (33.1)	106 (31.4)	115 (33.5)	114 (34.4)
Health insurance, n (%)				
Statutory	931 (92.0)	316 (93.5)	310 (90.4)	305 (92.1)
Private	81 (8.0)	22 (6.5)	33 (9.6)	26 (7.9)
Nationality, n (%)				
Non-German	33 (3.2)	14 (4.1)	10 (2.9)	9 (2.7)
German	979 (96.7)	324 (95.9)	333 (97.1)	322 (97.3)
Schooling degree^a^, n (%)				
None	14 (1.4)	6 (1.8)	4 (1.2)	4 (1.2)
Special needs education	4 (0.4)	3 (0.9)	0 (0.0)	1 (0.3)
Lower secondary school leaving certificate	167 (16.5)	62 (18.3)	57 (16.6)	48 (14.5)
Intermediate secondary school leaving certificate	207 (20.5)	68 (20.1)	70 (20.4)	69 (20.9)
Higher education entrance qualification for university of applied sciences	112 (11.1)	37 (10.9)	33 (9.6)	42 (12.7)
General higher education entrance qualification	508 (50.2)	162 (47.9)	179 (52.2)	167 (50.5)
Baseline total costs^b^^,c^(€), mean (SD)	4528 (10,734)^c^	4560 (8675)	4821 (11,672)	4192 (11,607)
EQ-5D^d^ index (−0.661 to 1), mean (SD)	0.69 (0.25)	0.70 (0.26)	0.69 (0.23)	0.68 (0.26)
EQ VAS^e^ (0‐100), mean (SD)	57.44 (21.93)	56.79 (21.89)	57.37 (22.36)	58.17 (21.57)
PHQ-9^f^ at baseline, mean (SD)	14.77 (4.00)	14.84 (4.21)	14.76 (4.00)	14.70 (3.79)

a Schooling degrees were translated based on the German Qualifications Framework [33].

b Baseline costs were assessed for a 6-mo preintervention period.

c A currency exchange rate of €1=US $1.02 was applicable as of December 31, 2022.

d EQ-5D-5L: EuroQol-5D-5L.

e EQ VAS: EuroQol Visual Analogue Scale

f PHQ-9: 9-item Patient Health Questionnaire.

The mean EQ-5D index score was 0.69 (SD 0.25), and the mean EQ VAS score was 57.44 (SD 21.93)—both scores being remarkably reduced compared to the German general population [34]. The mean total costs in the 6 months before the intervention were approximately €4528 (SD €10,734). Differences in baseline characteristics between all study arms were statistically not significant (Multimedia Appendix 4).

### Base Case Analysis

#### Costs During Follow-Up

Costs were highest for the nontailored feedback group and lowest for the tailored feedback group, with no statistically significant differences between study arms (Table 2). Overall, direct costs constituted approximately 64% of total costs, with inpatient costs making up the largest share (34%) of direct costs. All differences were statistically not significant.

**Table 2.** Mean costs, quality-adjusted life years (QALYs), and depression-free days (DFDs) during follow-up.

Category^a^	No feedback, mean (SD)	Nontailored feedback, mean (SD)	Tailored feedback, mean (SD)
Direct costs (€)	2860 (7555)	3316 (7727)	2549 (7444)
Inpatient services (€)	950 (4223)	1206 (5213)	795 (5337)
Outpatient physician services (€)	611 (1359)	684 (1388)	733 (3388)
Outpatient psychotherapist services (€)	321 (880)	389 (1030)	304 (798)
Outpatient nonphysician services (€)	169 (543)	163 (490)	143 (380)
Formal nursing care (€)	61 (923)	71 (713)	79 (643)
Informal care (€)	439 (2429)	587 (4256)	329 (2192)
Medication (€)	309 (3167)	216 (1171)	165 (850)
Indirect costs (€)	1776 (4312)	1693 (4131)	1490 (3543)
Total costs (€)	4636 (8953)	5008 (9241)	4039 (8408)
QALYs^b^	0.3636 (0.1030)	0.3620 (0.1111)	0.3575 (0.1110)
DFDs^c^	65.9 (51.3)	66.5 (50.5)	70.2 (49.0)

a A currency exchange rate of €1=US $1.02 was applicable as of December 31, 2022.

b Estimated based on the EuroQol-5D-5L index.

c Estimated based on 9-item Patient Health Questionnaire.

#### Quality of Life, QALY, and DFD During Follow-Up

In all study arms, the mean EQ-5D index scores as well as EQ VAS scores improved over the 6 months following the intervention, and the mean PHQ-9 scores decreased. Six months after the intervention, 9% (93/1012) of all participants reported *minimal* depression severity, and 33% (333/1012) had *mild*, 31% (317/1012) had *moderate*, 18% (179/1012) had *moderately severe*, and 9% (90/1012) had *severe* depressive symptoms.

Descriptive results (Table 2) indicated that the tailored feedback group experienced 0.3575 QALY during the posttreatment period, whereas nontailored feedback and no feedback experienced slightly more. The tailored feedback group experienced the most DFD, followed by nontailored feedback and no feedback. Differences in QALYs as well as DFDs were not statistically significant.

#### ICER and CEAC

As costs in the nontailored feedback group were higher (€372; *P=.*60) and QALYs were lower (−0.002; *P=*.85) compared to the no feedback group, the nontailored feedback group was dominated by the no feedback group. The tailored feedback group had both lower costs (€−970; *P=*.19*)* and lower effects (QALY −0.004; *P*=.60) than the nontailored feedback group, yielding an ICER of €216,873 per QALY. Compared to the no feedback group, the tailored feedback group had an ICER of €98,763 per QALY while also experiencing fewer QALY at lower costs (€−597; *P=*.40; and QALY −0.006; *P=*.48)*.*

**Figure 1.**
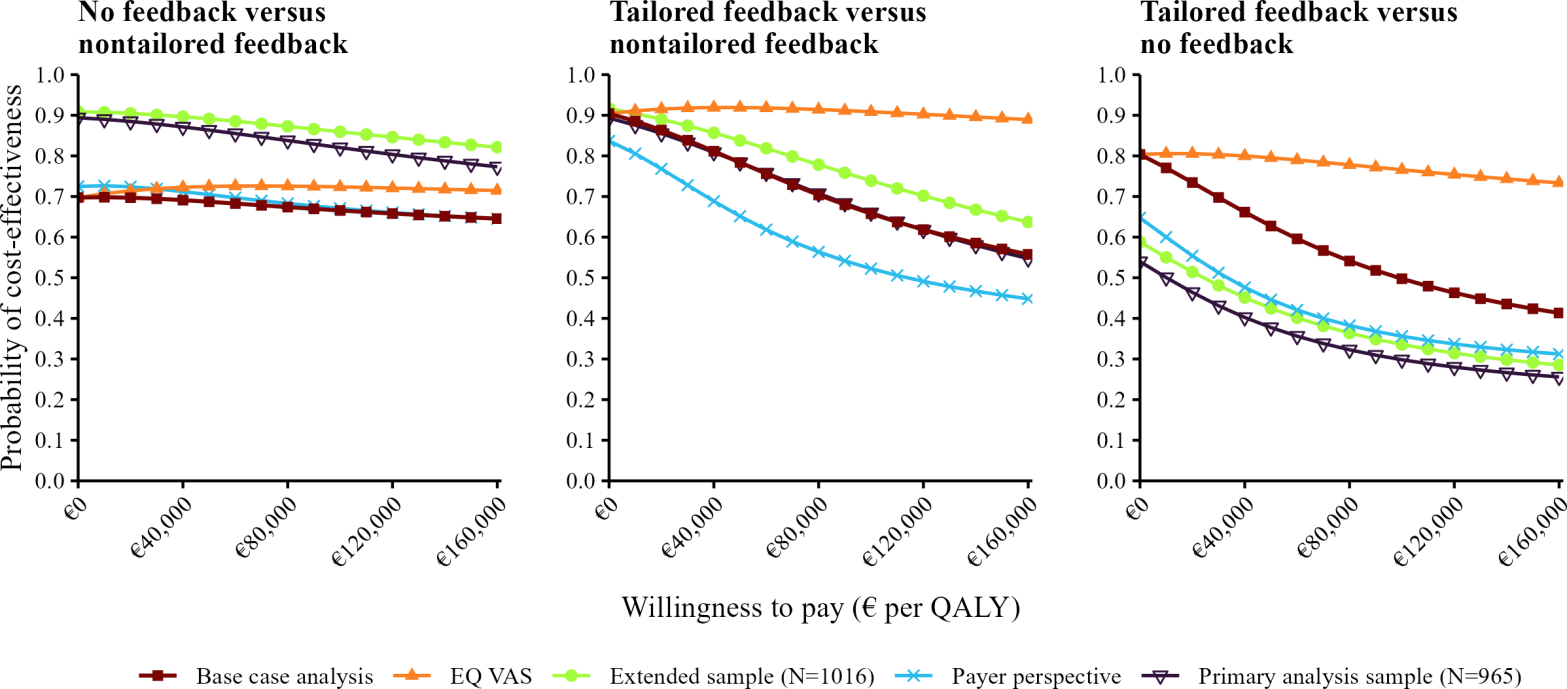
Cost-effectiveness acceptability curves. A currency exchange rate of €1=US $1.02 was applicable as of December 31, 2022. EQ VAS: EuroQol Visual Analogue Scale; QALY: quality-adjusted life year.

CEACs revealed cost-effectiveness probabilities between 70% (€0 per QALY) and 65% (€160,000 per QALY) for the no feedback group compared to the nontailored feedback group. For the tailored feedback group compared to the nontailored feedback group, CEACs revealed cost-effectiveness probabilities between 56% and 90%. Tailored feedback compared to no feedback revealed cost-effectiveness probabilities of 80% at a WTP margin of €0 per QALY and 41% at €160,000 per QALY (Figure 1).

### Sensitivity Analyses

The results from sensitivity analyses exhibited similar trends to the base case results (Figure 1). When considering QALY calculated from the EQ VAS (S1), both no feedback and nontailored feedback yielded higher cost-effectiveness probabilities compared to nontailored feedback. For the extended sample (S2), both no feedback and tailored feedback yielded higher cost-effectiveness probabilities when compared to the nontailored feedback arm. Cost-effectiveness probabilities in S2 were lower when comparing tailored feedback to no feedback. When taking a payer perspective (S3), cost-effectiveness probabilities were slightly lower when comparing tailored feedback to the other feedback options. When conducting the analysis for the sample used in the analysis of clinical effectiveness (S4), no feedback and tailored feedback yielded cost-effectiveness probabilities similar to those from the analysis of the extended dataset (S2).

**Figure 2.**
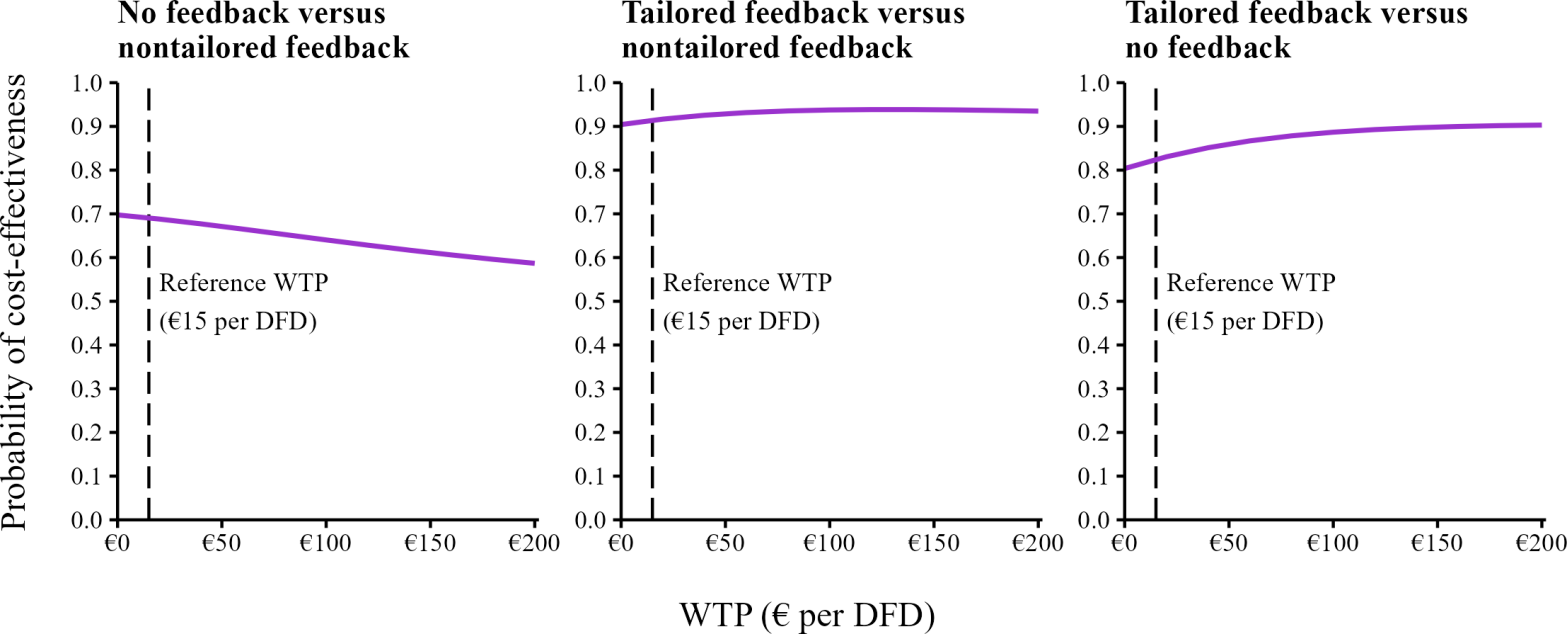
Cost-effectiveness acceptability curves considering depression-free days (DFD) as effect measure. A currency exchange rate of €1=US $1.02 was applicable as of December 31, 2022. WTP: willingness to pay.

Considering DFD as the effect measure (S5), participants in the tailored feedback arm experienced more DFD than both other study arms; however, differences were statistically not significant (Multimedia Appendix 4). Tailored feedback yielded high cost-effectiveness probabilities when compared to both nontailored feedback (90%‐94%) and no feedback (80%‐90%). No feedback compared to nontailored feedback exhibited cost-effectiveness probabilities decreasing from 70% (€0 per DFD) to 59% (€200 per DFD; Figure 2).

### Explorative Subgroup Analyses

The results of the CEAC for the subgroup analyses are shown in Multimedia Appendix 5. These analyses revealed that the tailored feedback intervention is more cost-effective for women than for men. Cost-effectiveness probabilities on baseline depression severity and age were inconclusive. The tailored intervention yielded the highest cost-effectiveness probabilities for participants whose suspected depression was confirmed using the Structured Clinical Interview for DSM-5 Disorders in a telephone interview, approximately 2 to 5 days after the intervention. Cost-effectiveness probabilities ranged between 93% (€0 per QALY) and 47% (€160,000 per QALY) when comparing tailored feedback to no feedback and between 80% and 87% when comparing it to nontailored feedback.

Tailored feedback had the lowest cost-effectiveness probabilities compared to no feedback for participants living in big cities (>100,000 inhabitants) and for participants not believing at baseline that they had depression.

## Discussion

### Principal Findings

This health economic evaluation has shown that nontailored feedback after web-based depression screening is associated with low cost-effectiveness probabilities, both when compared to no feedback and to tailored feedback.

In the following section, we will thus mainly focus on the comparison between tailored feedback and no feedback. Costs during follow-up did not significantly increase due to the automated nature of feedback interventions. HRQL in terms of QALY did not differ significantly between study arms, which is in line with differences in depression severity also being not statistically significant [13]. As all differences between study arms were statistically not significant and the ICER is a point estimate, we additionally constructed CEACs based on monetary NBR. In this approach, effect differences affect results more strongly than cost differences with increasing WTP, thus leading to a negatively sloped CEAC in the case of both negative costs and effect differences. Our results strongly depend on the assumed WTP margin, where tailored feedback has the highest cost-effectiveness probabilities at WTP thresholds below €20,000 per QALY.

DEPSCREEN-INFO is the only other RCT to date on feedback interventions in depression screening, for which a CEA was conducted [35,36]. In the trial, patients with heart disease were recruited, representing an older and predominantly male sample. The feedback intervention, which consisted of delivering the screening result to the patient in addition to their treating physician, yielded high cost-effectiveness probabilities and revealed significant improvements in depression severity 6 months after the intervention [35,36]. The high cost-effectiveness probability shown for the at-risk sample of DEPSCREEN-INFO could not be supported by the DISCOVER results [35]. Both the screening strategy (at risk vs general) and the delivery mode (in person vs internet-based) differed between DEPSCREEN-INFO and DISCOVER, which might explain the low cost-effectiveness probabilities in this CEA. A deeper analysis of the extent to which the delivery mode and the presence of depression risk factors potentially influence cost-effectiveness remains to be investigated.

This highlights the relevance of the recently conducted GET.FEEDBACK.GP RCT, which featured patient-targeted feedback and general practitioner (GP)-targeted feedback after depression screening in a primary care setting [37]. The clinical effectiveness study of GET.FEEDBACK.GP revealed no significant reduction in depression severity due to feedback interventions after depression screening for the general sample [37]. However, some subgroups exhibited potential beneficial effects postintervention [37]. The health economic analysis of GET.FEEDBACK.GP will provide further insights on the economic implications of delivering in-person feedback to the patient and the GP.

Of the 5 conducted scenario analyses, 3 showed similarly low cost-effectiveness probabilities of nontailored feedback compared to the other 2 study arms.

The scenarios considering a different effect measure, however, exhibited higher and more consistent cost-effectiveness probabilities of tailored feedback. The EQ VAS scenario yielded moderately high cost-effectiveness probabilities (73%‐80%) of tailored compared to screening only. When considering the disease-specific outcome DFD based on PHQ-9 scores, cost-effectiveness probabilities of tailored feedback were high (80%‐90%) compared to no feedback. Thus, automatically tailored feedback is likely to be cost-effective when considering the depression-specific measure DFD. This might be explained by the different types of patient-reported outcome measures considered in each analysis [38]. The EQ-5D-5L assesses overall functional status, and HRQL is estimated by applying preference-based value sets to the responses. The PHQ-9, on the other hand, assesses the depression-specific symptom status, a less complex construct than HRQL, by applying psychometric methods.

Explorative subgroup analyses revealed populations that might require other solutions than feedback after internet-based depression screening. Low cost-effectiveness probabilities of tailored feedback compared to screening only were most prominent for men, people living in big cities, people whose suspected depression was not confirmed using a diagnostic interview, and those who did not believe at baseline that they had a depressive disorder. On the other hand, moderately high to high cost-effectiveness probabilities of tailored feedback were observed for women, people living in medium-sized cities (20,000‐99,999 inhabitants), and people being diagnosed with depression shortly after screening.

As the study arms were not randomized with regard to these categories, the results need to be handled with care; however, this might provide an interesting starting point for further research. Furthermore, the question of whether participants believed that they had depression could be included in the randomization strategy, to gain more robust insights in future trials on depression screening combined with feedback interventions.

### Strengths and Limitations

In the DISCOVER trial, direct and indirect costs were assessed during follow-up and at baseline, allowing for differences between study arms to be tested, not only during follow-up. We noticed no statistically significant differences in all categories of interests, including costs, between study arms at baseline. Randomization can hence be considered successful, as the study arms were balanced and the sample was highly representative of a population interested in depression [13]. Additionally, this work is the first to estimate the health economic implications of feedback interventions after web-based depression screening.

However, this study has some limitations. The economic evaluation has the same methodological limitations as the analysis of the primary outcome, which included the absence of a no screening study group, the reliance on self-reports, and the self-selection into the trial [13].

We identified additional limitations in this CEA. First, data quality in the study may have been affected by the lengthiness of the questionnaires regarding the use of health care service use and the relatively short follow-up time horizon of 6 months. We expect this limitation to have influenced participants in all study arms similarly. Second, recall bias could have caused the underestimation of medical service use during the follow-up. We consider the risk of recall bias low, as the mean direct costs during follow-up were higher than in the comparable age group in the German population [39]. Third, QALYs were calculated based on the generic EQ-5D-5L in the base case analysis, which, on the one hand, is considered acceptably responsive for changes in anxiety and depression and, on the other hand, only considers mental health specifically in 1 of 5 dimensions and therefore tends to be less sensitive to changes in depression [40]. Fourth, the tailored feedback intervention might have increased participants’ awareness of their own health status and confronted them with their possible diagnosis. Minor symptoms, thus, could have been perceived more strongly, leading to a decrease in self-reported HRQL [41]. These limitations were handled by conducting S2, focusing on the depression-specific outcome of DFD calculated based on the PHQ-9.

### Conclusions

This CEA indicates that nontailored feedback after internet-based depression is less cost-effective compared to tailored feedback and depression screening without the provision of screening results. The cost-effectiveness of the tailored feedback intervention compared to no feedback highly depends on the assumed willingness-to-pay. Considering DFDs as an alternative disease-specific measure, the tailored feedback intervention demonstrates consistently high cost-effectiveness probabilities. In this case, it could be considered economically reasonable compared to both other study arms. Nevertheless, it is important to note that feedback interventions did not significantly impact costs, HRQL, or depression severity throughout the follow-up period. Further research is needed to identify optimal target populations and the most effective methods to present screening results.

## Supplementary material

10.2196/68282 Multimedia Appendix 1 Participants flow diagram.

10.2196/68282 Multimedia Appendix 2 Overview of incorporated costs per sector.

10.2196/68282 Multimedia Appendix 3 Comparison of different sample sizes.

10.2196/68282 Multimedia Appendix 4 Baseline group comparison of study population.

10.2196/68282 Multimedia Appendix 5 Cost-effectiveness acceptability curves for subgroup analyses.

## Abbreviations

CEA: cost-effectiveness analysis
CEAC: cost-effectiveness acceptability curve
DFD: depression-free day
EQ VAS: EuroQol Visual Analogue Scale
EQ-5D-5L: EuroQol-5D-5L
GP: general practitioner
HRQL: health-related quality of life
ICER: incremental cost-effectiveness ratio
NBR: net-benefit regression
PHQ-9: 9-item Patient Health Questionnaire
QALY: quality-adjusted life year
RCT: randomized controlled trial
WTP: willingness-to-pay
