# Supplementary figures and images for: Automated Feedback After Internet-Based Depression Screening: Cost-Effectiveness Analysis of a Randomized Controlled Trial
Source: JMIR Form Res. 2025 Dec 23;9:e68282. doi: 10.2196/68282 (PMC12724478; doi:10.2196/68282)

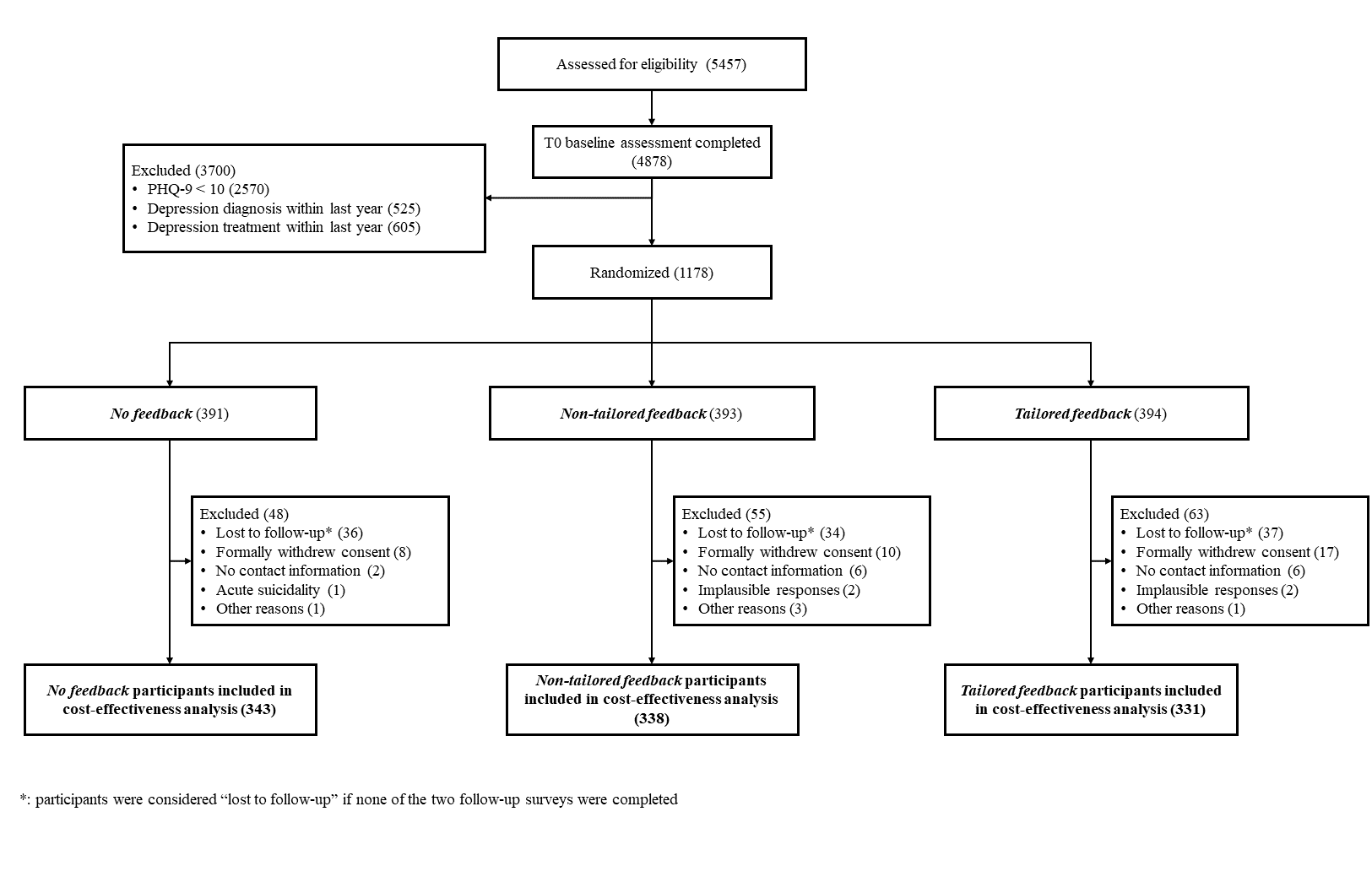
**Multimedia Appendix 1: Participants flow diagram**

Supplement: Multimedia Appendix 1 [file formative-v9-e68282-s001.docx]

**Multimedia Appendix 5: Cost-effectiveness acceptability curves for subgroup analyses.**


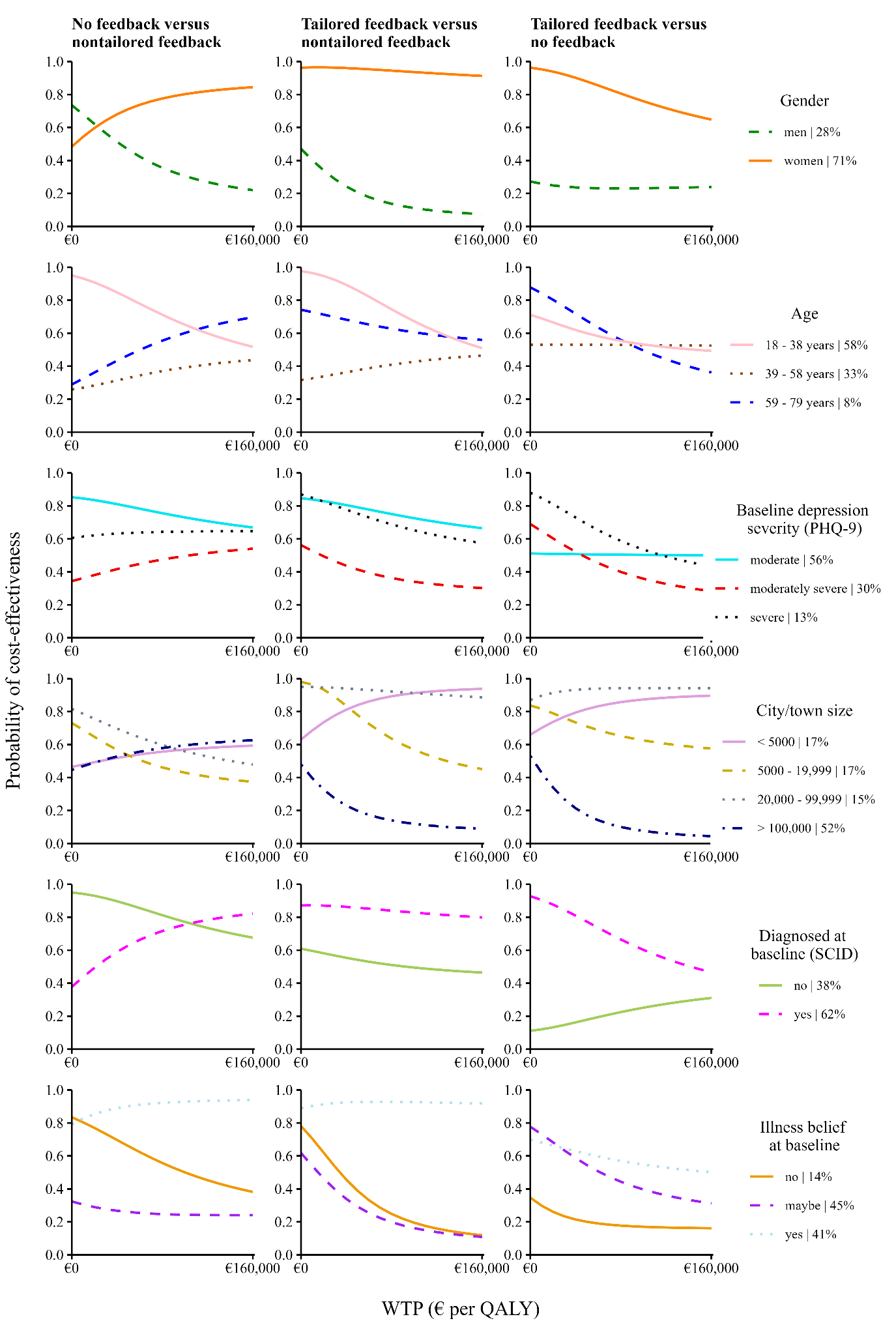

Supplement: Multimedia Appendix 5 [file formative-v9-e68282-s005.docx]
