# Supplementary material for: Automated Feedback After Internet-Based Depression Screening: Cost-Effectiveness Analysis of a Randomized Controlled Trial
Source: JMIR Form Res. 2025 Dec 23;9:e68282. doi: 10.2196/68282 (PMC12724478; doi:10.2196/68282)
Supplement: Multimedia Appendix 2 [file formative-v9-e68282-s002.docx]

**Multimedia Appendix 2: Overview of incorporated costs per sector.**

| **Sector** | **Services / Goods** | **Unit** | **Unit costs** |
| --- | --- | --- | --- |
| Inpatient services | General, psychiatric and rehabilitation hospitals (inpatient and partly inpatient) | Day | Type specific mean rates^a^ |
| Outpatient physician services | GP, specialists (e.g. internist, cardiologist, gynecologist, etc.) | Contact | Type specific mean rates^a,b,c,d^ |
| Outpatient non-physician services | e.g. physiotherapy, speech therapy, massage, etc. | Contact | Reimbursement schedule^a^ |
| Outpatient psychotherapy | Psychotherapist | Contact | Reimbursement schedule^a^ |
| Medication | Product (e.g. pills, tablets, ointments, drops, etc.) | Quantity | Official pharmaceutical index^e^ |
| Nursing care | Professional nursing care | Minute | Type specific wage^a^ |
|  | Informal care | Hour | Type specific wage (substitution cost approach)^a^ |
| Indirect costs | Productivity losses (paid work) | Hour | Gross income plus nonwage labor costs^f,g^ |

a: Muntendorf L-K, Brettschneider C, Konnopka A, König H-H. [Updating standardized unit costs from a societal perspective for health economic evaluation] Aktualisierung der standardisierten Bewertungssätze aus gesellschaftlicher Perspektive für gesundheitsökonomische Evaluationen. *Gesundheitswesen* 2024; (EFirst).

b: Bock JO, Brettschneider C, Seidl H, Bowles D, Holle R, Greiner W, König HH. Calculation of Standardised Unit Costs from a Societal Perspective for Health Economic Evaluation. *Gesundheitswesen* 2015; **77**(01): 53–61.

c: PECUNIA Group. PECUNIA Reference Unit Costs Compendium (PECUNIA RUC Compendium). Zenodo; 2021.

d: vdek Hamburg. Hamburger Rettungsdienst: Gebühren steigen um 15 Prozent. 2021. <https://www.vdek.com/LVen/HAM/fokus/Rettungsdienst/RettungsdienstKosten.html> (accessed March 07, 2023).

e: RLS GmbH. Rote Liste 2022, Q1. Frankfurt/Main: Rote Liste Service GmbH; 2008.

f: Statistisches Bundesamt (Destatis). Durchschnittliche Bruttomonatsverdienste, Zeitreihe. 2023. <https://www.destatis.de/DE/Themen/Arbeit/Verdienste/Verdienste-Branche-Berufe/Tabellen/liste-bruttomonatsverdienste.html?nn=206824> (accessed March 07, 2023).

g: Statistisches Bundesamt (Destatis). Wöchentliche Arbeitszeit: Deutliche Unterschiede in der EU. 20.09.2022 2022. <https://www.destatis.de/Europa/DE/Thema/Bevoelkerung-Arbeit-Soziales/Arbeitsmarkt/Wochenarbeitszeiten.html?nn=217388> (accessed March 07, 2023).
