# Supplementary material for: Automated Feedback After Internet-Based Depression Screening: Cost-Effectiveness Analysis of a Randomized Controlled Trial
Source: JMIR Form Res. 2025 Dec 23;9:e68282. doi: 10.2196/68282 (PMC12724478; doi:10.2196/68282)
Supplement: Multimedia Appendix 3 [file formative-v9-e68282-s003.docx]

**Multimedia Appendix 3: Comparison of different sample sizes.**

| **Characteristics** | **Base case sample**  **N = 1,012** | **Primary analysis sample**  **N_primary_ = 965** | | **Extended sample**  **N_ext_ = 1,016** | | **Comparison method** |
| --- | --- | --- | --- | --- | --- | --- |
|  |  |  | **p-value^a^** |  | **p-value^a^** |  |
| **Age [years]** |  |  | *.90* |  | *.95* | Mann-Whitney *U* test |
| mean (SD) | 37.50 (14.09) | 37.39 (14.04) |  | 37.54 (14.11) |  |  |
| **Gender: %** |  |  | *.20* |  | *.20* | Chi-squared test |
| Female | 70.75 | 70.88 |  | 70.57 |  |  |
| Male | 28.36 | 28.19 |  | 28.54 |  |  |
| Diverse | 0.89 | 0.93 |  | 0.89 |  |  |
| **Living situation: %** |  |  | *>.99* |  | *>.99* | Chi-squared test |
| With someone | 66.90 | 66.94 |  | 66.93 |  |  |
| Alone | 33.10 | 33.06 |  | 33.07 |  |  |
| **Health insurance: %** |  |  | *>.99* |  | *>.99* | Chi-squared test |
| Statutory | 92.00 | 92.12 |  | 92.03 |  |  |
| Private | 8.00 | 7.88 |  | 7.97 |  |  |
| **Nationality: %** |  |  | *>.99* |  | *>.99* | Chi-squared test |
| Non-German | 3.26 | 3.32 |  | 3.35 |  |  |
| German | 96.74 | 96.68 |  | 96.65 |  |  |
| **Schooling degree: %** |  |  | *.22* |  | *.22* | Chi-squared test |
| None | 1.38 | 1.24 |  | 1.38 |  |  |
| Special education | 0.40 | 0.41 |  | 0.39 |  |  |
| *Mittelschule* | 16.50 | 15.75 |  | 16.63 |  |  |
| *Mittlere Reife* | 20.45 | 20.31 |  | 20.37 |  |  |
| *Fachabitur* | 11.07 | 10.98 |  | 11.12 |  |  |
| *Abitur* | 50.20 | 51.30 |  | 50.10 |  |  |
| **Baseline total costs^b^ [€]** |  |  | *.71* |  | *.91* | Mann-Whitney *U* test |
| mean (SD) | 4,528 (10,734) | 5,110 (16,546) |  | 5,058 (16,217) |  |  |
| **EQ-5D index (-0.661 – 1)** |  |  | *.87* |  | *.89* | Mann-Whitney *U* test |
| mean (SD) | 0.69 (0.25) | 0.69 (0.25) |  | 0.69 (0.25) |  |  |
| **EQ VAS (0 – 100)** |  |  | *.92* |  | *.97* | Mann-Whitney *U* test |
| mean (SD) | 57.44 (21.93) | 57.53 (21.80) |  | 57.46 (21.96) |  |  |
| **PHQ-9 at baseline** |  |  | *.91* |  | *.96* | Mann-Whitney *U* test |
| mean (SD) | 14.77 (4.00) | 14.77 (3.96) |  |  |  |  |
| ^a^ p-values for the primary analysis sample and the extended sample are displayed against the base case sample,  ^b^ Baseline costs were assessed for a 6-months pre-intervention period  SD: Standard deviation; PHQ-9: Patient Health Questionnaire 9 | | | | | |  |
