# Supplementary material for: Automated Feedback After Internet-Based Depression Screening: Cost-Effectiveness Analysis of a Randomized Controlled Trial
Source: JMIR Form Res. 2025 Dec 23;9:e68282. doi: 10.2196/68282 (PMC12724478; doi:10.2196/68282)
Supplement: Multimedia Appendix 4 [file formative-v9-e68282-s004.docx]

**Multimedia Appendix 4: Baseline group comparison of study population.**

| **Characteristics** | **Non-tailored feedback**  **n = 338** | **No feedback**  **n = 343** | | **Tailored feedback**  **n = 331** | | |
| --- | --- | --- | --- | --- | --- | --- |
|  |  |  | ***P* value^d^** |  | ***P* value^d^** | ***P* value^e^** |
|  |  |  |  |  |  |  |
| **Age [years]** |  |  | .24^a^ |  | .37^a^ | .79^a^ |
| mean (SD) | 38.25 (14.04) | 36.98 (13.74) |  | 37.27 (14.49) |  |  |
| **Gender: %** |  |  |  |  |  |  |
| Female | 70.12 | 72.30 |  | 69.79 |  |  |
| Male | 28.99 | 26.82 | .53^c^ | 29.31 | .93^c^ | .47^c^ |
| Diverse | 0.89 | 0.87 | .96^c^ | 0.91 | .98^c^ | .93^c^ |
| **Living situation: %** |  |  |  |  |  |  |
| With someone | 68.64 | 66.47 | .55^b^ | 65.56 | .40^b^ | .80^b^ |
| Alone | 31.36 | 33.53 |  | 34.44 |  |  |
| **Health insurance: %** |  |  |  |  |  |  |
| Statutory | 93.49 | 90.48 | .14^b^ | 92.15 | .50^b^ | .42^b^ |
| Private | 6.51 | 9.62 |  | 7.85 |  |  |
| **Nationality: %** |  |  |  |  |  |  |
| Non-German | 4.14 | 2.92 | .39^b^ | 2.72 | .32^b^ | .88^b^ |
| German | 95.86 | 97.08 |  | 97.28 |  |  |
| **Schooling degree: %** |  |  |  |  |  |  |
| None | 1.78 | 1.17 |  | 1.21 |  |  |
| Special education | 0.89 | 0.00 | .98^c^ | 0.30 | .60^c^ | .98^c^ |
| *Mittelschule* | 18.34 | 16.62 | .63^c^ | 14.50 | .82^c^ | .81^c^ |
| *Mittlere Reife* | 20.12 | 20.41 | .52^c^ | 20.85 | .53^c^ | .98^c^ |
| *Fachabitur* | 10.95 | 9.62 | .67^c^ | 12.69 | .44^c^ | .75^c^ |
| *Abitur* | 47.93 | 52.19 | .44^c^ | 50.45 | .51^c^ | .92^c^ |
| **Baseline total costs^f^ [€]** |  |  | .75^a^ |  | .66^a^ | .45^a^ |
| mean (SD) | 4,560 (8,675) | 4,821 (11,672) |  | 4,192 (11,607) |  |  |
| **EQ-5D index (-0.661 – 1)** |  |  | .65^a^ |  | .32^a^ | .58^a^ |
| mean (SD) | 0.70 (0.26) | 0.69 (0.23) |  | 0.68 (0.26) |  |  |
| **EQ VAS (0 – 100)** |  |  | .73^a^ |  | .42^a^ | .64^a^ |
| mean (SD) | 56.79 (21.89) | 57.37 (22.36) |  | 58.17 (21.57) |  |  |
| **PHQ-9 at baseline** |  |  | .80^a^ |  | .65^a^ | .85^a^ |
| mean (SD) | 14.84 (4.21) | 14.76 (4.00) |  | 14.70 (3.79) |  |  |
| ^a^ Linear regression, ^b^ Binary logistic regression, ^c^ Multinomial logistic regression, ^d^ non-tailored feedback as reference level, ^e^ no feedback as reference level  ^f^ Baseline costs were assessed for a 6-months pre-intervention period  SD: Standard deviation; PHQ-9: Patient Health Questionnaire 9 | | | | | | |
